# Machine Learning to Predict Faricimab Treatment Outcome in Neovascular Age-Related Macular Degeneration

**DOI:** 10.1016/j.xops.2023.100385

**Published:** 2023-08-18

**Authors:** Yusuke Kikuchi, Michael G. Kawczynski, Neha Anegondi, Ales Neubert, Jian Dai, Daniela Ferrara, Carlos Quezada-Ruiz

**Affiliations:** 1 Roche Personalized Healthcare Program, Genentech, Inc., South San Francisco, California; 2 Department of Industrial Engineering and Operations Research, University of California, Berkeley, Berkeley, California; 3 Clinical Imaging Group, Genentech, Inc., South San Francisco, California; 4 Data & Analytics, Roche Pharma Research and Early Development, Basel, Switzerland; 5 Clinical Science, Genentech, Inc., South San Francisco, California; 6 Department of Ophthalmology, Clínica de Ojos Garza Viejo, San Pedro Garza, Garcia, Nuevo Leon, Mexico

**Keywords:** Machine learning, Optical coherence tomography, Neovascular age-related macular degeneration, Faricimab, Treatment

## Abstract

**Purpose:**

To develop machine learning (ML) models to predict, at baseline, treatment outcomes at month 9 in patients with neovascular age-related macular degeneration (nAMD) receiving faricimab.

**Design:**

Retrospective proof of concept study.

**Participants:**

Patients enrolled in the phase II AVENUE trial (NCT02484690) of faricimab in nAMD.

**Methods:**

Baseline characteristics and spectral domain-OCT (SD-OCT) image data from 185 faricimab-treated eyes were split into 80% training and 20% test sets at the patient level. Input variables were baseline age, sex, best-corrected visual acuity (BCVA), central subfield thickness (CST), low luminance deficit, treatment arm, and SD-OCT images. A regression problem (BCVA) and a binary classification problem (reduction of CST by 35%) were considered. Overall, 10 models were developed and tested for each problem. Benchmark classical ML models (linear, random forest, extreme gradient boosting) were trained on baseline characteristics; benchmark deep neural networks (DNNs) were trained on baseline SD-OCT B-scans. Baseline characteristics and SD-OCT data were merged using 2 approaches: model stacking (using DNN prediction as an input feature for classical ML models) and model averaging (which averaged predictions from the DNN using SD-OCT volume and from classical ML models using baseline characteristics).

**Main Outcome Measures:**

Treatment outcomes were defined by 2 target variables: functional (BCVA letter score) and anatomical (percent decrease in CST from baseline) outcomes at month 9.

**Results:**

The best-performing BCVA regression model with respect to the test coefficient of determination (R^2^) was the linear model in the model-stacking approach with R^2^ of 0.31. The best-performing CST classification model with respect to test area under receiver operating characteristics (AUROC) was the benchmark linear model with AUROC of 0.87. A post hoc analysis showed the baseline BCVA and the baseline CST had the most effect in the all-model prediction for BCVA regression and CST classification, respectively.

**Conclusions:**

Promising signals for predicting treatment outcomes from baseline characteristics were detected; however, the predictive benefit of baseline images was unclear in this proof-of-concept study. Further testing and validation with larger, independent datasets is required to fully explore the predictive capacity of ML models using baseline imaging data.

**Financial Disclosure(s):**

Proprietary or commercial disclosure may be found in the Footnotes and Disclosures at the end of this article.

Age-related macular degeneration (AMD) is a leading cause of vision loss in patients 50 years of age and older and presents in 2 advanced clinical forms: neovascular AMD (nAMD) and geographic atrophy.^1^^,^^2^ Neovascular AMD is characterized by choroidal neovascularization (also called macular neovascularization) and is associated with vision loss that, if not properly treated in a timely manner, can be irreversible.^2^ Standard of care for nAMD in the past 15 years has been anchored in intravitreal injections of anti-VEGF agents, administered monthly, bimonthly, and every 3 months with a treat-and-extend regimen, or on a *pro re nata* (as needed) strategy.^3^, ^4^, ^5^ However, data from clinical practice show a notable contrast between visual gains and outcomes achieved in the pivotal phase III anti-VEGF trials in comparison with those seen in the clinical setting, which has been attributed, among other causes, to undertreatment and the broad variability in treatment frequency.^6^^,^^7^ In addition, outcomes reported in clinical trials represent the “average” patient cohort response rather than the individualized response of each patient over time,^8^ whereas broad heterogeneity in the response to treatment is commonly seen in nAMD.^9^

Recently, innovative therapeutic options for nAMD have been made available, including faricimab.^10^ This is the first bispecific antibody designed for intraocular use that blocks both angiopoietin-2 and VEGF-A, 2 growth factors thought to play key roles in the pathogenesis of nAMD and other retinal vascular diseases.^11^ Faricimab’s clinical development plan included 1 phase I (NCT01941082) and 2 phase II trials in nAMD (AVENUE [NCT02484690] and STAIRWAY [NCT03038880]) in which faricimab was found to be well-tolerated and achieve vision and anatomical outcomes comparable with intravitreal anti-VEGF monotherapy.^12^, ^13^, ^14^ In the phase III TENAYA (clinicaltrials.gov; NCT03823287) and LUCERNE (NCT03823300) trials, faricimab administered up to every 16 weeks led to improved visual acuity outcomes that were noninferior to aflibercept, a treatment that targets the VEGF pathway alone, administered every 8 weeks.^15^ Given the variable treatment response in nAMD in clinical practice and the increasing number of treatment options, the ability to predict future individual treatment outcomes at baseline could support drug development and help clinicians make personalized treatment decisions, potentially improving patient outcomes while reducing treatment burden.

Artificial intelligence–based tools, including machine learning (ML), could potentially represent an innovative and complementary approach to address these unmet needs in the current management of nAMD as well as in future drug development. Machine learning–based algorithms have demonstrated the potential to identify baseline prognostic factors^8^ and predict treatment response to or requirements for anti-VEGF agent using visual or clinical characteristics and anatomical imaging data taken at the baseline or first few months of treatment.^9^

In this proof-of-concept study, we aimed to explore whether ML using baseline clinical variables and baseline spectral domain-OCT (SD-OCT) could predict future treatment outcomes for faricimab-treated eyes with nAMD in the AVENUE trial.

## Methods

### Source of Data

**Figure 1:**
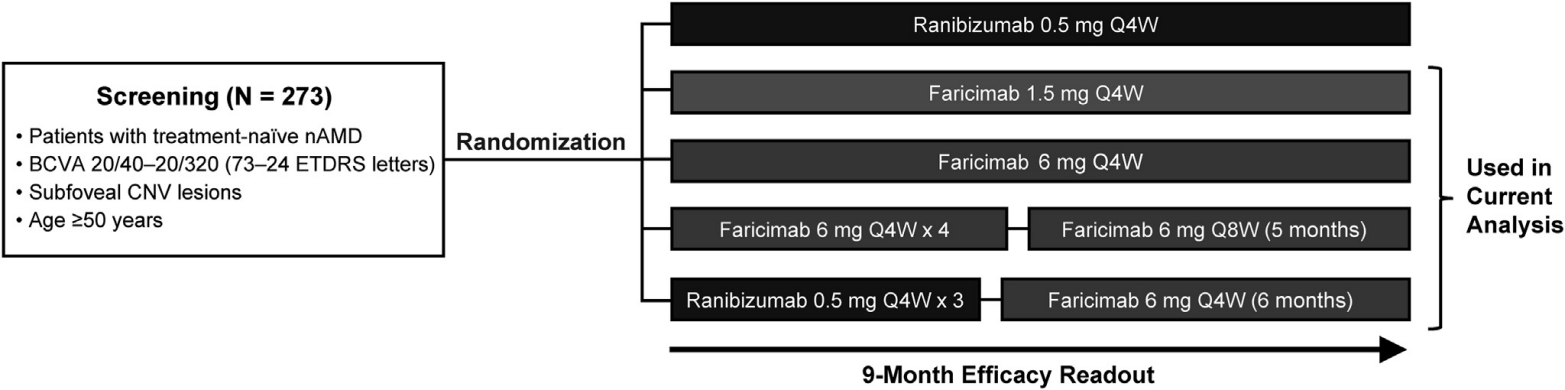
The AVENUE trial design, with the 4 treatment arms included in the current analysis indicated. BCVA = best-corrected visual acuity; CNV = choroid neovascularization; nAMD = neovascular age-related macular degeneration; Q4W = every 4 weeks; Q8W = every 8 weeks.

The AVENUE trial was a double-masked, 36-week, multicenter, comparator-controlled, parallel group phase II randomized trial in treatment-naive patients with subfoveal choroidal neovascularization secondary to nAMD.^14^ The trial was conducted in accordance with the Declaration of Helsinki, principles of Good Clinical Practice, and in compliance with applicable local laws; protocols were approved by the applicable institutional review boards.^14^ Written informed consent was obtained from all patients. The AVENUE trial comprised 5 treatment arms (Fig 1), including the comparator arm (ranibizumab dosed every 4 weeks), which was excluded from the current analysis. Only 1 eye per patient was selected as the study eye and was included in the study. Details of the trial design and results have been previously published.^14^

**Figure 2:**
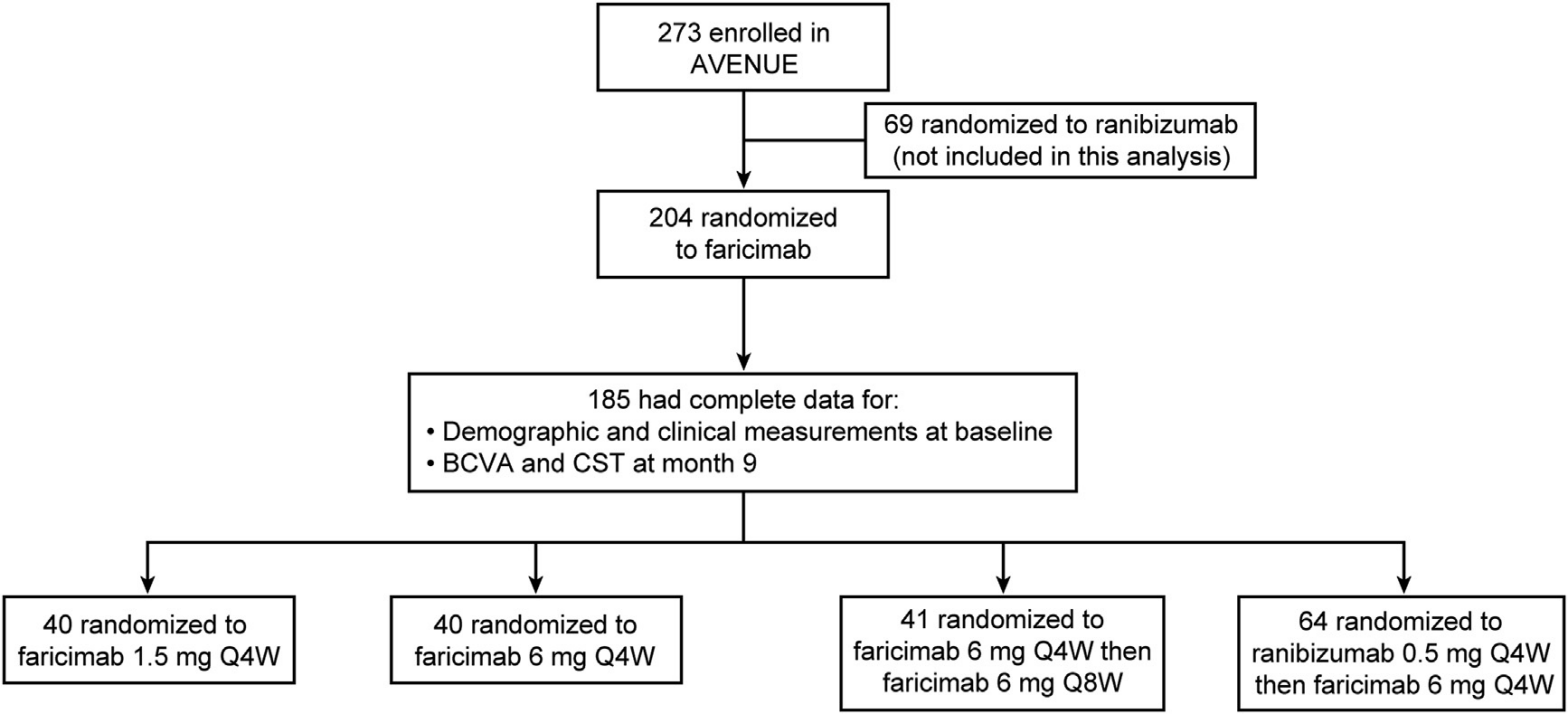
The AVENUE trial patient disposition for patients included in the present analysis.^a^^a^Full patient disposition for the AVENUE trial has been previously published.^14^ BCVA = best-corrected visual acuity; CST = central subfield thickness; Q4W = every 4 weeks; Q8W = every 8 weeks.

Of 273 patients enrolled in the AVENUE trial, 204 were randomized to the 4 faricimab treatment arms. Of these, 185 patients (185 study eyes) had both complete data for demographic and clinical measurements at day 1 (baseline) and complete data for best-corrected visual acuity (BCVA) and for SD-OCT central subfield thickness (CST) at month 9 (Fig 2; Table 1). These 185 patients with the complete dataset of interest were included in the current study.

**Table 1:** Patient Demographics and Characteristics at Baseline and Month 9 in Patients Included in This Analysis from the AVENUE Trial

	Baseline (N = 185)	Month 9 (N = 185)
Age, mean (SD) [range], years	78.5 (8.77) [55, 96]	–
Sex, n		–
Male	60	
Female	125	
Treatment arm, n		–
Arm B	40	
Arm C	40	
Arm D	41	
Arm E	64	
BCVA, mean (SD) [range], ETDRS letter score	55.6 (12.1) [24, 83]	63.4 (16.1) [14, 89]
CST, mean (SD) [range], μm	461 (131) [230, 899]	287 (71.1) [188, 602]
LLD, ETDRS letter score, mean (SD) [range]	20.3 (9.9) [0, 43]	–
Percent decrease in CST from baseline, mean (SD) [range], μm	–	0.341 (0.206) [−0.656, 0.692]

Arm B = faricimab 1.5 mg every 4 weeks; Arm C = faricimab 6.0 mg every 4 weeks; Arm D = faricimab 6.0 mg every 4 weeks to week 12, followed by every 8 weeks; Arm E = ranibizumab 0.5 mg every 4 weeks to week 8, followed by faricimab 6.0 mg every 4 weeks; BCVA = best-corrected visual acuity; CST = central subfield thickness; LLD = low luminance deficit; SD = standard deviation.

### Outcome Variables and Fold Definitions

In this study, treatment outcomes were defined as either functional or anatomical.

The functional outcome was defined as the BCVA letter score at month 9 (the primary outcome measure in the AVENUE trial). For the functional outcome prediction, a regression problem was considered. The coefficient of determination (R^2^) score was used as the primary metric to evaluate model performance; root mean squared error and mean absolute error were used as secondary metrics to assess the performance from different aspects.

The anatomical outcome was defined by the percent decrease in CST from baseline to month 9. The percent decrease in CST from baseline was converted to a binary variable (i.e., a variable with only true/false values) with a threshold of 35%, which was chosen based on the median percent decrease in CST from baseline of 36.5% observed in the dataset. Thus, the binary variable can be broadly interpreted as whether a given individual patient exhibited a reduction in CST greater than the median reduction observed for all patients in the trial. For the anatomical outcome prediction, the primary metric was area under the receiver operator characteristic (AUROC) curve; secondary metrics were accuracy, precision, and recall. The closest point to the top left corner in the receiver operator characteristics plot was chosen as the operating point, or threshold, for accuracy, precision, and recall.

The entire dataset was split at the patient level into 80% (148 patients) training and 20% (37 patients) test sets. The training set was further divided into 5 folds of equal size to perform cross-validation (CV). All splits were stratified by the target variable (quartile for BCVA regression).

### Input Variables

Two types of input variables were considered at baseline to predict the outcome variables: tabular data and image data. Tabular data are characterized by the fact that the value has an actual meaning of clinical relevance. Image data do not fall into the category of tabular data because the meaningful information in an image is the global structure of the object shown and the value of each individual pixel is less meaningful.

#### Tabular Data

The following tabular variables at baseline were included: age (years), sex, baseline BCVA letter score, CST (μm), low luminance deficit, and treatment arm. Central subfield thickness was defined as the average thickness between the inner limiting membrane (ILM) and the retinal pigment epithelium over the central 1 mm subfield. Low luminance deficit was defined as the difference between BCVA and low luminance visual acuity. The distribution of tabular data is shown in Table 1.

#### Image Data

Image data consisted of the macular SD-OCT images at baseline. All SD-OCT images from study eyes were taken using Spectralis (Heidelberg Engineering, Inc., Heidelberg, Germany; Table 2). The SD-OCT image acquisition protocol varied across patients and sites, and volumetric SD-OCT data could include 19, 36, 47, or 49 B-scans. In patients imaged with 19 B-scans, the distance between each B-scan was twice that of the B-scans taken in other patients.

**Table 2:** Summary of Spectral Domain-OCT B-Scans Taken

Patients, n	Number of Scans Taken	Dimension of B-Scan (Pixels)	Area Covered by Each Scan (mm^2^)
158	49	496 × 512	2 × 6
25∗	19	496 × 758	2 × 4.5
1	47	496 × 512	2 × 6
1	36	496 × 512	2 × 4

∗ In these 25 patients, the distance between each B-scan was twice that of the B-scans taken in other patients.

**Figure 3:**
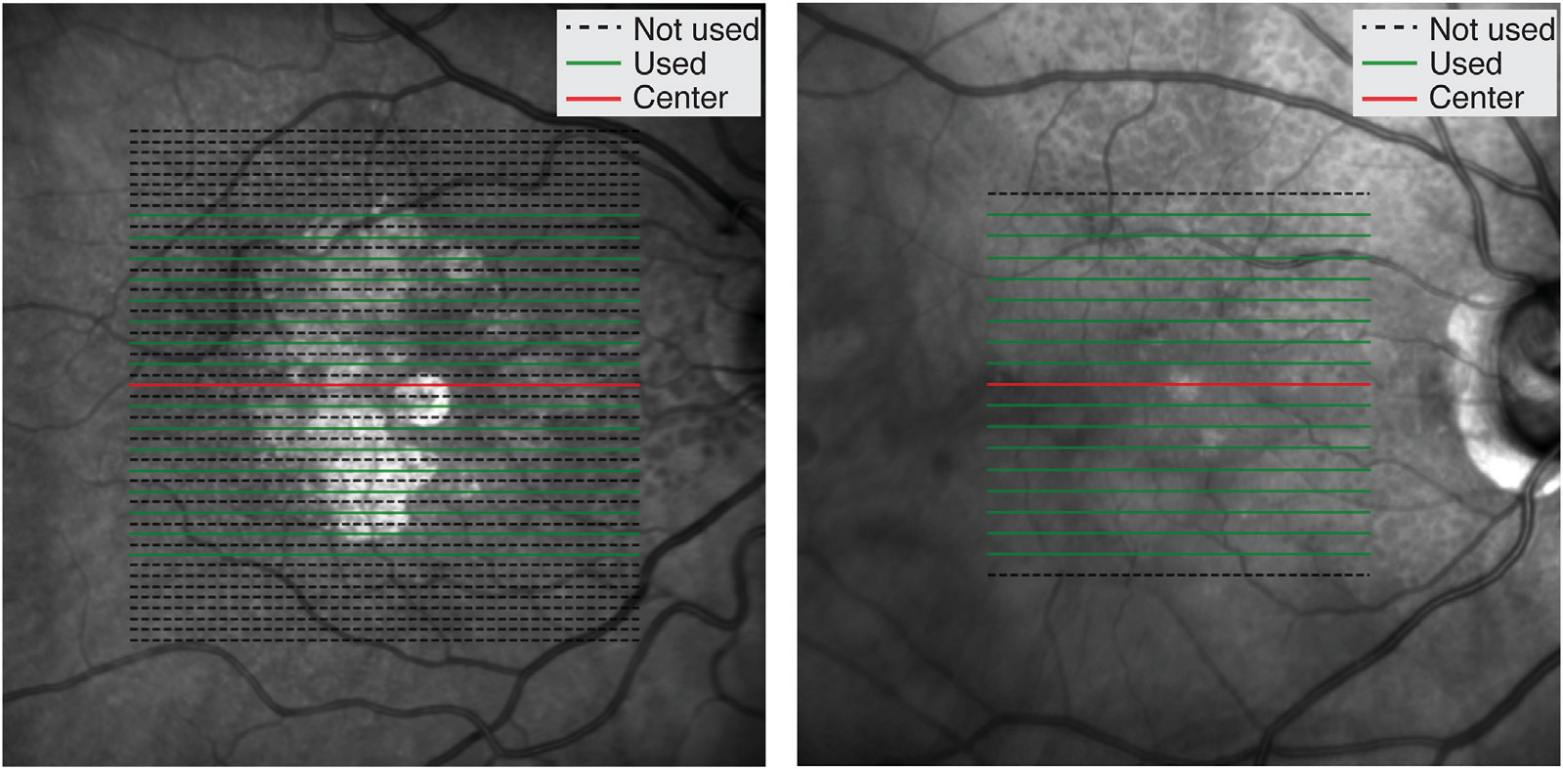
Representation of B-scans selected for spectral domain-OCT reprocessing, according to the number of scans in each volume. (Left) OCT volume with 49 B-scans, (Right) OCT volume with 19 B-scans.

**Figure 4:**
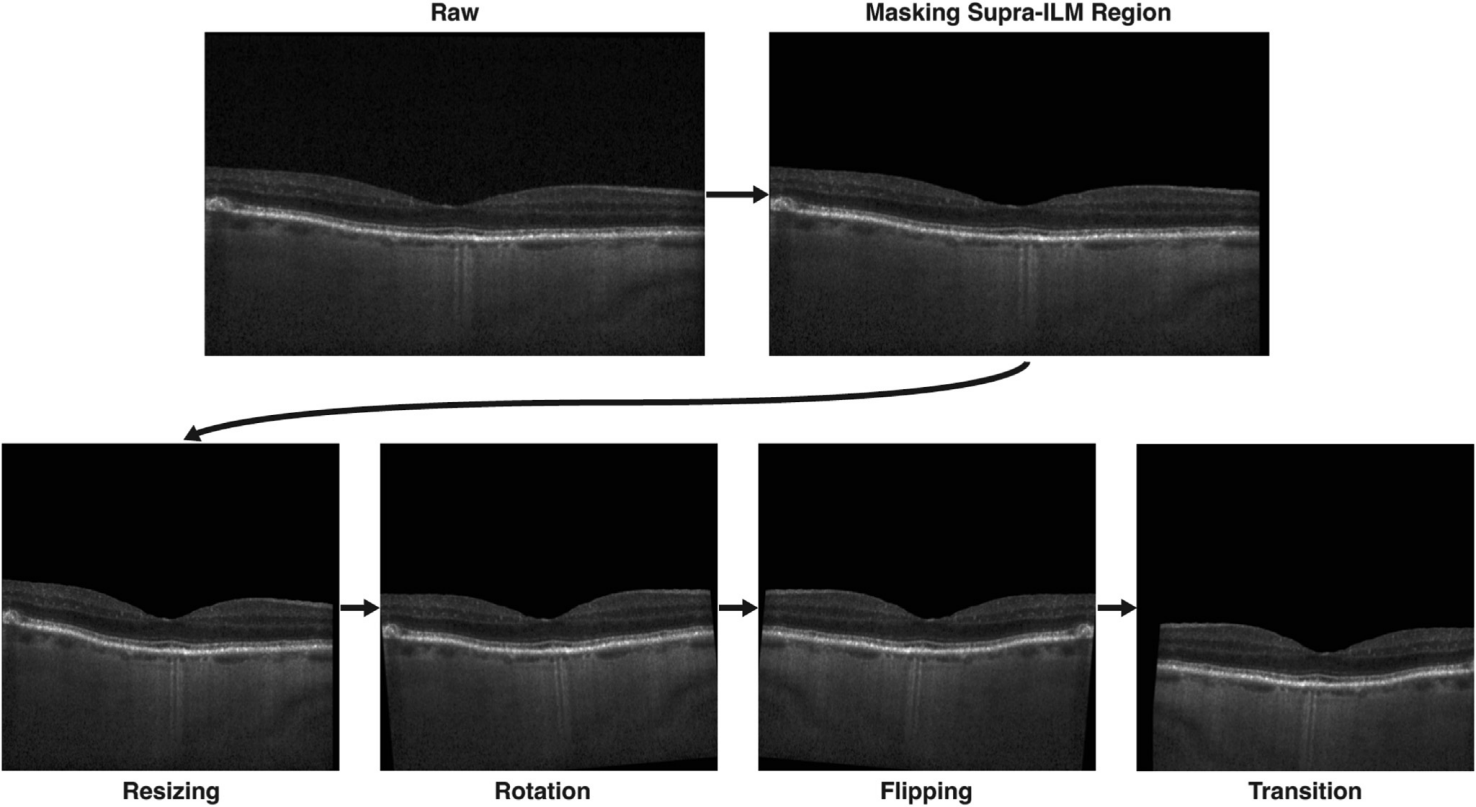
Image augmentation process. Rotation, flipping, and translation are applied randomly in the training. For the presented example, rotation of 5 degrees, flipping, vertical translation of 50 pixels, and horizontal translation of 25 pixels were applied. Augmentations shown here were applied for the purpose of illustration. ILM = inner limiting membrane.

For SD-OCT preprocessing, all B-scans were resampled to the same pixel resolution and resized to 496 × 512 pixels using bilinear interpolation. Seventeen B-scans from approximately the same location were included for each SD-OCT volume; the central or foveal B-scan was always included, and 8 B-scans were taken from each side of the central B-scan. Because SD-OCT volumes with 19 B-scans had twice the distance between consecutive B-scans than other volumes, the 16 noncentral B-scans were taken alternately in SD-OCT volumes with 49, 47, or 36 B-scans (Fig 3). The suprainner limiting membrane (the region above the inner limiting membrane or the region of vitreous in the B-scan) was masked (the pixel values were set to 0). To complement the small sample size, the following data augmentation techniques were applied to training images: random rotation (no rotation, rotation of 5 degrees, rotation of −5 degrees), random left/right flip, and random translation (vertical translation and horizontal translation are uniformly distributed over [−50, 50] and [−25, 25] in pixels, respectively) (Fig 4).

### Benchmark ML Models

Two types of models were used corresponding to the 2 types of input data. The classical ML models took the tabular data as input data, and the deep neural networks (DNNs) processed the SD-OCT image data. The classical ML models and the DNNs were used as the benchmark models because they consisted of a single ML algorithm, whereas the model-stacking and averaging approaches used 2 algorithms. The results of the classical ML models were used to see whether improvement can be seen in the model-stacking and averaging approaches. The model performance of the deep learning (DL) model was used to understand how well image information was extracted.

#### Classical ML Models

To explore models with different learning mechanisms, we included the following 3 models: linear (elastic net), random forest (RF), and extreme gradient boosting (XGBoost^16^) models, whose hyperparameters were tuned in the 5-fold CV. For each of the 3 models, 2 instances were developed: 1 for the BCVA letter score regression and 1 for the percent decrease in CST from baseline classification; therefore, a total of 6 classical ML models were developed. All classical ML models were implemented using the Scikit-learn module in Python.^17^

#### DL Model

Deep leaning is a field of ML that uses DNNs. A DNN learns useful patterns for a prediction in the given dataset by itself without being manually programmed. The DL model only took SD-OCT images as input data. The average of predictions for 17 B-scans from the same SD-OCT volume was used for patient-level prediction. The base architecture for the model was Inception version 3,^18^ which is a DNN for image processing. A global average pooling layer and a dropout layer were inserted before the output layer. The output layer was regularized with L1 penalty. The DL algorithms were implemented using TensorFlow^19^ and Keras^20^ in Python. Again, 2 separate instances were developed for each of the 2 prediction problems.

ImageNet^21^ pretrained weights were used as the initial point of training. The training details common to both BCVA regression and percent decrease in CST from baseline classification (except where explicitly noted) were as follows: the model was trained with the Adam optimizer^22^ with a learning rate of 10^−6^ using batches of 8 SD-OCT images for 180 epochs. Dropout was applied after average pooling and before the output layer during training with 0.95 probability. The L1 penalty coefficient was 0.1 for BCVA regression and 0.05 for percent decrease in CST from baseline classification. For training of the BCVA model, mean squared loss was used, and for training of the CST model, binary cross-entropy loss was used.

#### Model-Stacking and Model-Averaging Approaches

The classical ML models and the DL model only used either the tabular or the SD-OCT image data. We proposed 2 approaches to combine tabular data and SD-OCT image data: model stacking and model averaging.

**Figure 5:**
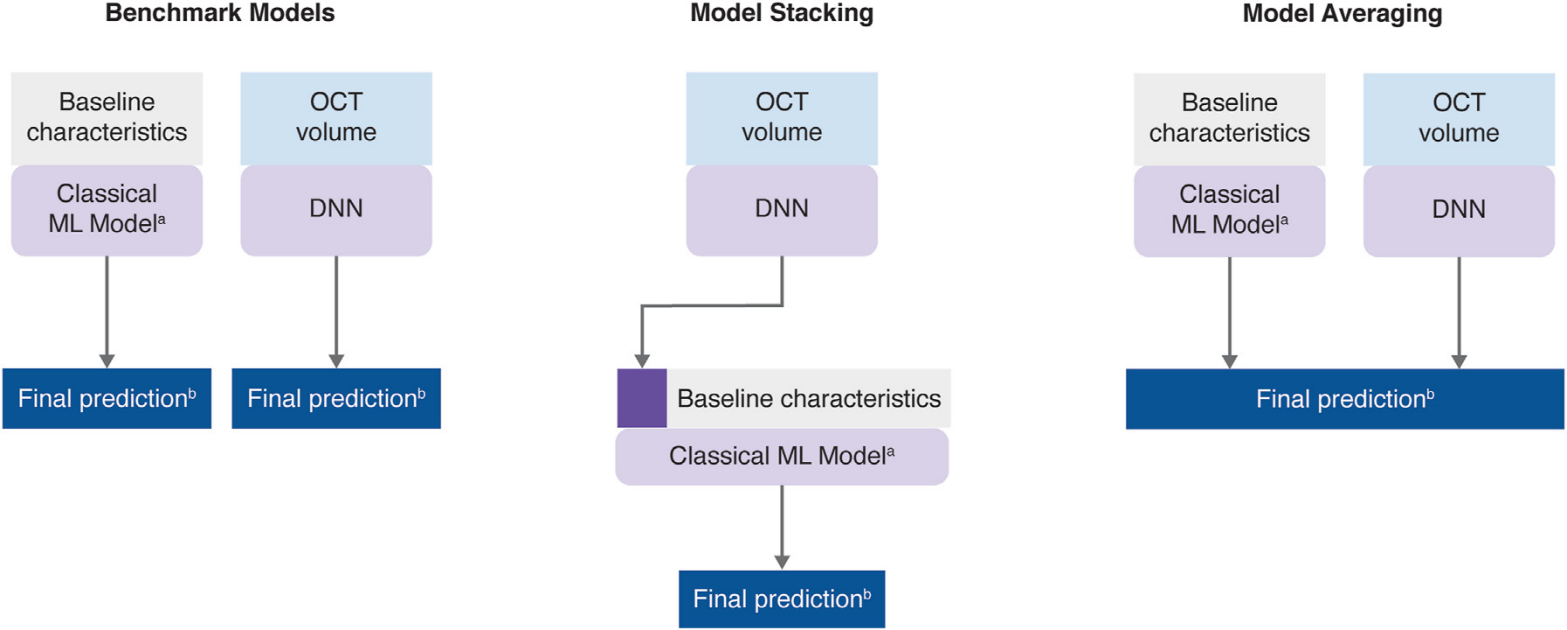
Overview of the benchmark models, model-stacking, and model-averaging. Baseline characteristics are tabular data. ^a^Classical ML models are either linear model, random forest, or XGBoost. ^b^Final prediction is either BCVA at month 9 or the probability of having >35% decrease in CST from baseline. BCVA = best-corrected visual acuity; CST = central subfield thickness; DNN = deep neural network; ML = machine learning; XGBoost = extreme gradient boosting.

Model-stacking^23^ involved a 2-stage approach. At the first stage, the DL model was trained, and the resulting prediction was used as 1 of the input features into the classical ML model at the second stage (Fig 5). For the first stage CV, 5-fold CV was used to tune hyperparameters of the DL models. In iteration *i* (*i* = 1, 2, 3, 4, 5) of the second stage 5-fold CV, the prediction of the DL model from iteration *i* of the first stage CV was used as 1 of the input features. Six models were developed using the model-stacking approach.

In the model-averaging approach, the classical ML models and DL models were trained separately. The final prediction was the (equally weighted) average of the classical ML model prediction (on tabular data) and the DL model prediction (on SD-OCT data; Fig 5). Again, 6 models were developed using the model-averaging approach.

### Testing Procedure

To calculate the test metrics, classical ML models were retrained on the entire training dataset with the optimal hyperparameters found in 5-fold CV. DL models were used in an ensemble way (i.e., the average of 5 DL models [from each 5-fold CV iteration] was used).

The methods used to examine the impact of clinical features on outcome variables are summarized in the Supplement (available at https://www.ophthalmologyscience.org).

## Results

### CV Metrics

**Figure 6:**
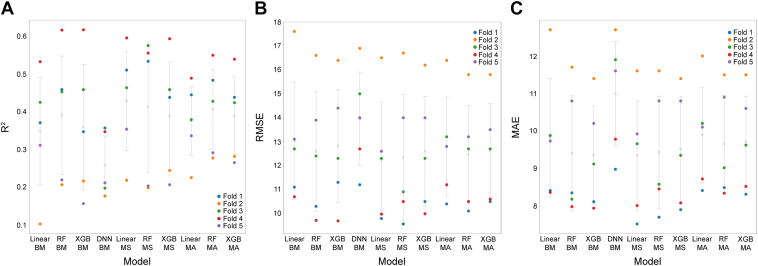
Cross-validation (CV) results of best-corrected visual acuity regression, for (**A**) R^2^ scores in CV, (**B**) root mean squared error in CV, and (**C**) mean absolute error (MAE) in CV. Crosses represent the mean score of 5 folds; error bars represent the standard deviation of 5 folds; circles represent the metric score for each fold. BM = benchmark; DNN = deep neural network; MA = model averaging; MS = model stacking; R^2^ = coefficient of determination; RF = random forest; RMSE = root mean squared error; XGBoost = extreme gradient boosting.

The CV results for BCVA regression are shown in Figure 6. The R^2^ (standard deviation [SD]) values of benchmark classical ML models, whose inputs are tabular data were 0.35 (0.14) for linear model, 0.39 (0.16) for RF, and 0.36 (0.17) for XGBoost. The image-based DNN model had a mean R^2^ (SD) value of 0.26 (0.08). In BCVA regression, for the functional treatment outcome prediction, model-stacking and model-averaging showed improvements in CV metrics compared with the benchmark classical ML models, with a larger improvement seen with model-stacking (Fig 6); this was apparent across the linear, RF, and XGBoost models. The highest R^2^ value was observed with model-stacking in the linear model (0.43 [0.13]).

**Figure 7:**
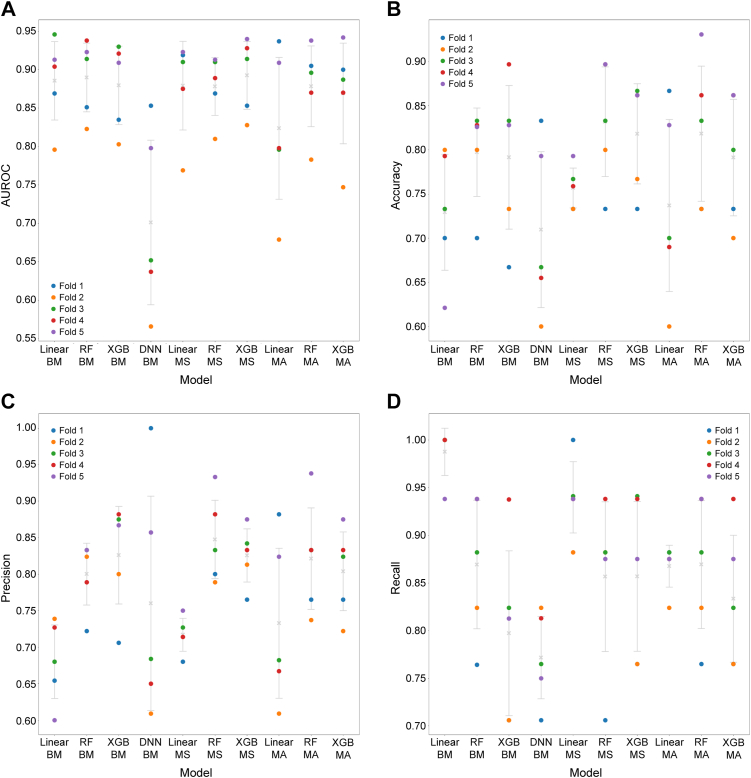
Cross-validation (CV) results of percent decrease in central subfield thickness from baseline classification, for (**A**) area under the receiver operator characteristic curve (AUROC) in CV, (**B**) accuracy in CV, (**C**) precision in CV, and (**D**) recall in CV. Crosses represent the mean score of 5 folds; error bars represent the standard deviation of 5 folds; circles represent the metric score for each fold. BM = benchmark; DNN = deep neural network; MA = model averaging; MS = model stacking; RF = random forest; XGBoost = extreme gradient boosting.

For the anatomical treatment outcome prediction, the CV results for percent decrease in CST from baseline classification (threshold of 35%) are shown in Figure 7. The mean (SD) AUROC of the benchmark classical ML models were 0.89 (0.05) for the linear model, 0.89 (0.04) for RF, and 0.88 (0.05) for XGBoost. The result for DNN was a mean AUROC (SD) of 0.77 (0.11). No clear improvement was observed in CV metrics between models with respect to benchmark classical ML models, model-stacking, and model-averaging (Fig 7). The highest mean AUROC value was 0.89 (0.04) for the XGBoost model with model-stacking, and the highest accuracy value was 0.87 (0.05) for the RF model with model-stacking.

### Test Metrics

**Figure 8:**
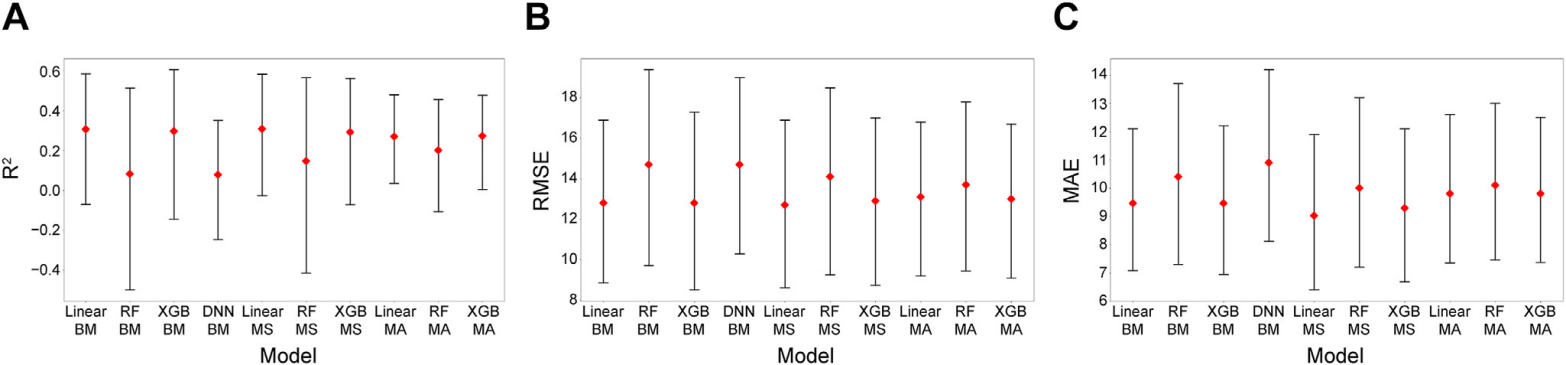
Test results of best-corrected visual acuity regression, for (**A**) coefficient of determination (R^2^) scores in cross-validation (CV), (**B**) root mean squared error (RMSE) in CV, and (**C**) mean absolute error (MAE) in CV. Diamonds represent the metric calculated on the test set; error bars represent 95% confidence intervals (bootstrap number = 1000). BM = benchmark; DNN = deep neural network; MA = model averaging; MS = model stacking; R^2^ = coefficient of determination; RF = random forest; XGBoost = extreme gradient boosting.

For the functional treatment outcome prediction, considering BCVA regression, the benchmark linear model, RF, and XGBoost had R^2^ values of 0.31, 0.08, and 0.30, respectively. The DNN showed an R^2^ value of 0.08. The highest R^2^ value for a model-stacking or model-averaging instance was 0.31 for model-stacking with linear model (Table 3; Fig 8). The highest R^2^ value and lowest root mean squared error and mean absolute error values were observed with the linear model (model-stacking; Table 3). Overfitting was most prominent for the RF models.

**Table 3:** Test Results of Best-Corrected Visual Acuity Regression for Each Model

Model	R^2^ (95% CI)	RMSE (95% CI)	MAE (95% CI)
Benchmark models			
Linear	0.306 (−0.0702, 0.584)	12.8 (8.85, 16.9)	9.46 (7.08, 12.1)
Random forest	0.0825 (−0.499, 0.513)	14.7 (9.71, 19.4)	10.4 (7.29, 13.7)
XGBoost	0.297 (−0.143, 0.606)	12.8 (8.5, 17.3)	9.46 (6.95, 12.2)
Deep neural network	0.0786 (−0.246, 0.351)	14.7 (10.3, 19.0)	10.9 (8.12, 14.2)
Model stacking			
Linear	0.308 (−0.0261, 0.583)	12.7 (8.61, 16.9)	9.02 (6.39, 11.9)
Random forest	0.147 (−0.413, 0.566)	14.1 (9.26, 18.5)	10.0 (7.21, 13.2)
XGBoost	0.292 (−0.0708, 0.561)	12.9 (8.73, 17.0)	9.29 (6.68, 12.1)
Model averaging			
Linear	0.270 (0.0358, 0.479)	13.1 (9.20, 16.8)	9.80 (7.36, 12.6)
Random forest	0.201 (−0.107, 0.456)	13.7 (9.44, 17.8)	10.1 (7.46. 13.0)
XGBoost	0.273 (0.00396, 0.478)	13.0 (9.10, 16.7)	9.80 (7.37, 12.5)

CI = confidence interval; MAE = mean absolute error; R^2^ = coefficient of determination; RMSE = root mean squared error; XGBoost = extreme gradient boosting.

**Figure 9:**
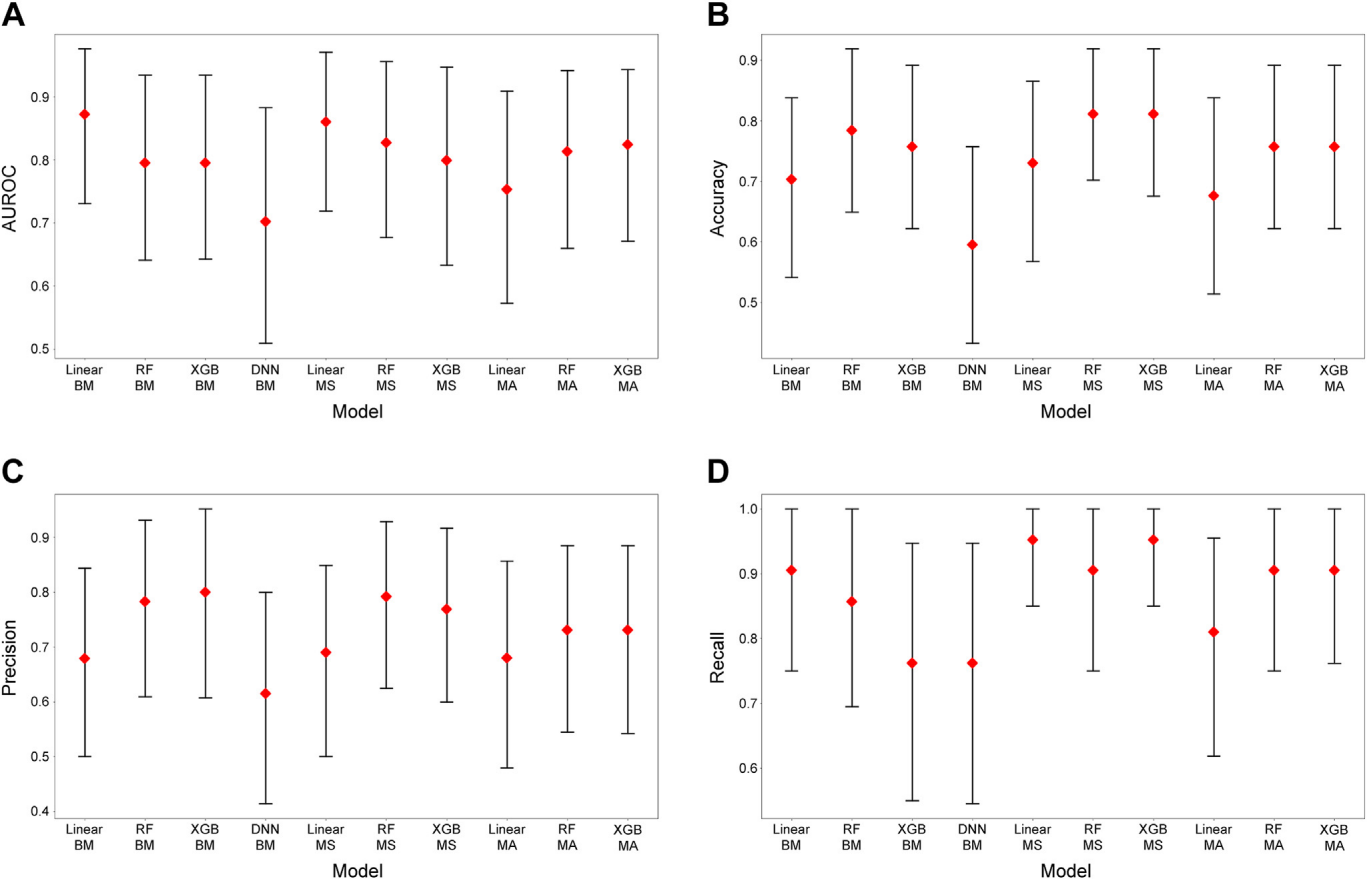
Test results of percent decrease in central subfield thickness from baseline classification, for (**A**) area under the receiver operator characteristic curve (AUROC) in cross-validation (CV), (**B**) accuracy in CV, (**C**) precision in CV, and (**D**) recall in CV. Diamonds represent the metric calculated on the test set; error bars represent 95% confidence intervals (bootstrap number = 1000). BM = benchmark; DNN = deep neural network; MA = model averaging; MS = model stacking; RF = random forest; XGBoost = extreme gradient boosting.

For percent decrease in CST from baseline classification, the benchmark linear model, RF, and XGBoost had AUROC values of 0.87, 0.80, and 0.80, respectively. The DNN had an AUROC value of 0.70. The highest AUROC value for a model-stacking or model-averaging instance was 0.86 for model-stacking with linear model, which makes the benchmark linear model the best-performing model (Table 4; Fig 9).

**Table 4:** Test Results of Central Subfield Thickness Reduction Rate Classification for Each Model

Model	AUROC (95% CI)	Accuracy (95% CI)	Precision (95% CI)	Recall (95% CI)
Benchmark models				
Linear	0.872 (0.731, 0.976)	0.838 (0.730, 0.976)	0.895 (0.762, 1.00)	0.810 (0.643, 1.00)
Random forest	0.795 (0.641, 0.934)	0.811 (0.676, 0.919)	0.792 (0.650, 0.958)	0.905 (0.619, 1.00)
XGBoost	0.795 (0.643, 0.934)	0.784 (0.676, 0.919)	0.760 (0.619, 0.950)	0.905 (0.654, 1.00)
Deep neural network	0.702 (0.509, 0.883)	0.757 (0.595, 0.892)	0.833 (0.650, 1.00)	0.714 (0.435, 0.941)
Model stacking				
Linear	0.860 (0.719, 0.971)	0.838 (0.730, 0.946)	0.800 (0.690, 1.00)	0.952 (0.632, 1.00)
Random forest	0.827 (0.677, 0.956)	0.811 (0.703, 0.946)	0.792 (0.654, 1.00)	0.905 (0.579, 1.00)
XGBoost	0.799 (0.633, 0.947)	0.838 (0.730, 0.946)	0.826 (0.666, 0.957)	0.905 (0.762, 1.00)
Model averaging				
Linear	0.753 (0.573, 0.909)	0.784 (0.649, 0.919)	0.842 (0.682, 1.00)	0.762 (0.480, 0.950)
Random forest	0.813 (0.660, 0.942)	0.784 (0.703, 0.919)	0.842 (0.652, 1.00)	0.762 (0.591, 1.00)
XGBoost	0.824 (0.671, 0.943)	0.784 (0.649, 0.919)	0.760 (0.636, 1.00)	0.905 (0.476, 1.00)

AUROC = area under the receiver operator characteristic curve; CI = confidence interval; XGBoost = extreme gradient boosting.

Results regarding the impact of clinical features in benchmark models and model-stacking approach on outcome variables were obtained from SHAP analysis (SHapley Additive exPlanations^24^) (Supplement Material; available at https://www.ophthalmologyscience.org). For BCVA regression, baseline BCVA was consistently found to be the most impactful feature. In the model-stacking approach, the prediction from DNN was found to be the second most important among linear model, RF, and XGBoost. The impact of the other features was mostly limited. On the other hand, for the CST classification, the baseline CST was found to be the most impactful feature throughout benchmark models and model-averaging approach. The baseline BCVA and DNN predictions were also found to be impactful on the prediction. The baseline BCVA was ranked as the second most important feature in the benchmark models and third most impactful in model-stacking approach, whereas the DNN prediction was second most impactful in all models in the model-stacking approach. Additionally, comparisons of the predictions with true data are summarized in the Supplement (available at https://www.ophthalmologyscience.org).

## Discussion

In this proof-of-concept study, benchmark models and 2 novel approaches, involving modeling multimodal inputs for predicting the treatment outcome to faricimab at month 9 in patients with nAMD from baseline data, were developed and evaluated systematically. Overall, our models suggest the potential of using ML-based algorithms to predict future treatment outcomes in this context. We discuss herein the interpretation of the results of the ML models presented in this study.

First, we evaluated benchmark classical ML models. For BCVA regression, the linear and XGBoost models achieved a test R^2^ score of approximately 0.3, which is consistent with a similar previous analysis of a DL model using OCT images to predict BCVA.^25^ The RF model in this study was overfitted to the training set, and the performance was rather unstable, as indicated by large confidence intervals. For the percentage decrease in CST from baseline classification, the linear benchmark model had the highest AUROC value of 0.87 and an accuracy of 0.84. All classical ML models showed significant predictive power, as demonstrated by the lower confidence interval being distant from 0.5.

The performance of the benchmark DL models was low for both BCVA regression and CST reduction classification. There are 3 possible reasons for this. The first is that the correlation between anatomical features detected by SD-OCT (e.g., CST) and functional features (e.g., those measured with BCVA) is weak, as previously shown.^26^^,^^27^ In fact, this weak correlation between specific imaging biomarkers on SD-OCT and visual function as measured by BCVA has been a matter of debate.^28^^,^^29^ Although this is more relevant to BCVA regression, it is also relevant to the CST reduction classification, as BCVA was an impactful feature for percent decrease in CST from baseline classification (Supplement Material; available at https://www.ophthalmologyscience.org) and the BCVA information cannot be interpreted entirely in SD-OCT imaging.

The second reason is likely the small sample size, which was a main limitation of this study. Although pretraining on ImageNet eased and stabilized the training process to some extent, there was still a large gap between the number of parameters and the sample size; Inception version 3 has approximately 24 million parameters, whereas the number of B-scans used to train each DL model was approximately 2000 (∼17 B-scan × 148 patients × 0.8; where 0.8 represents the portion of training samples in each CV iteration [4 folds for training]). Furthermore, 17 B-scans from the same volume typically looked similar to each other in regard to clinically relevant image biomarkers, which implies that the effective sample size was even smaller.

The final reason for low performance could be that our DL modeling approach lacks the learned interaction between B-scans. Our approach of simply taking the average of predictions on 17 B-scans may not be optimal because the central B-scans may be more informative than the off-center B-scans. An alternative approach would be to introduce the learned interaction by replacing the average with learned weights^30^ or even process the 3-dimensional OCT volume directly. In view of these additional complexities and given the small sample size, a simple modeling strategy was adopted in the present context of this initial proof of concept study.

We compared the results of the benchmark classical ML models with model-stacking and model-averaging. In the BCVA regression, model-stacking improved the performance of the linear and RF models from the corresponding benchmark results in all metrics, and model-averaging improved the performance of the RF model in all metrics. In terms of R^2^ score, the percentage improvement was 0.65% for the linear model with model-stacking; improvements for the RF model were 78% with model-stacking and 144% with the model-averaging approach. In fact, the model-stacking approach made the linear model the best-performing model for the regression problem.

In the CST reduction classification, improvements in AUROC (the primary metric) were observed for both model-stacking and model-averaging with the RF and XGBoost models, but not for the best-performing model, which was the benchmark linear model. Although the model-stacking and model-averaging approaches did not significantly improve the highest performing model, it did substantially increase the performance of those models with low performance in most cases. This observation suggests that the variance explained by the SD-OCT image data probably overlaps the variance explained by the tabular data, but when the relationship between the tabular data and the target variable was not learned well, the image data could complement it.

To summarize model performance, the linear model in model-stacking and the benchmark linear model were the best-performing models for BCVA regression and percent decrease in CST classification, respectively. The model-stacking and model-averaging approaches did not improve the best primary metrics, but they helped stabilize the model performance.

In addition to the model performance, the interpretability of the models is also a relevant concern in clinical applications. According to the SHAP analysis for the benchmark classical ML models and model-stacking approach, baseline BCVA, and baseline CST were consistently the most impactful features for BCVA regression and CST classification, respectively. This suggests that the level of the corresponding feature at baseline is strongly tied with future status after the treatments. Looking at the relationship between BCVA (functional) and CST (anatomical), we see asymmetry. Baseline CST was not impactful for the prediction of BCVA, but baseline BCVA had a coherent impact on CST classification. In the model-stacking approach, the model prediction was found to be impactful, but this should be interpreted with the caveats discussed next. Other features, including low luminescence deficit, which was reportedly predictive of the BCVA response for anti-VEGF therapy,^31^ were not found to be consistently impactful for the model prediction. The potential reason for this could be the small sample size, which hinders the ability to detect the signal from noise. Due to its low performance, we did not conduct interpretability analyses for the DL models.

Taking a closer look at the development of the model-stacking approach, we should highlight that the CV of the model stacking in this analysis used the same folds in the first and second stages. Consequently, there was an indirect information leakage, and the CV results of model stacking were optimistic. Although nested CV or using a holdout set for the second stage CV would be ideal, the aforementioned approach was justified in this case because (1) DL training cannot be run too many times due to limited computational resources and (2) the size of the training set was limited.

An end-to-end DL model was used to extract information from SD-OCT images. An alternative approach would be to use a segmentation model for known biomarkers using a list of potential predictors preselected by experts. The advantage of our approach is that the DL model may find new patterns or biomarkers by learning important features directly from the images by itself. In this sense, end-to-end DL models could lead to clinical and scientific insights unrestricted by the use of a priori selected biomarkers. However, in some cases, clinicians may find segmentation-based models using well-known image biomarkers more easily interpretable.

In summary, this is the first study to demonstrate that ML using baseline data can be used to predict specific functional and anatomical future treatment outcomes with faricimab, a new treatment option for patients with nAMD. Two methods were tested to merge the clinical tabular data and the SD-OCT imaging data. This proof-of-concept study was designed to test an ML approach and algorithm for predicting faricimab treatment outcomes in a small sample of nAMD patients; it was not intended to be a comprehensive study evaluating differences between treatment arms. Our modeling approach delivered moderately good predictive values despite limitations in the sample size. Results indicate that further studies are warranted to fully explore the predictive capability of the models with or without SD-OCT image data and to validate the presented methodology on a larger and independent dataset. With further improvement and validation of this emerging technology, these models could potentially help identify patient populations with certain characteristics or treatment requirements, which may be relevant for targeted drug development. Ultimately, these artificial intelligence–based approaches could enable clinicians to make timely and personalized treatment decisions for patients with nAMD to achieve the best possible outcomes for each patient.

## Data Sharing

For up-to-date details on Roche's Global Policy on the Sharing of Clinical Information and how to request access to related clinical study documents, see here: https://go.roche.com/data_sharing. For eligible studies, qualified researchers may request access to the clinical data through a data request platform. At the time of writing, this request platform is Vivli: https://vivli.org/ourmember/roche/. For the imaging data underlying this publication, requests can be made by qualified researchers, subject to a detailed, hypothesis-driven proposal and necessary agreements.

## Supplementary Data

Supplementary Materials

Figure S10 SHapley Additive exPlanations (SHAP) plots performed on the test set for best-corrected visual acuity regression at month 9 for (**A**) linear benchmark model, (**B**) random forest benchmark model, (**C**) extreme gradient boosting benchmark model, (**D**) linear model with model stacking, (**E**) random forest model with model stacking, and (**F**) extreme gradient boosting benchmark model with model stacking. Avg = average; CST = central subfield thickness; FAR = faricimab; LLD = low luminance deficit; M = male; Q4W = every 4 weeks; Q8W = every 8 weeks; RBZ = ranibizumab.

Figure S11 SHapley Additive exPlanations (SHAP) plots performed on the test set for percent decrease in central subfield thickness (CST) from baseline classification at month 9 for (**A**) linear benchmark model, (**B**) random forest benchmark model, (**C**) extreme gradient boosting benchmark model, (**D**) linear model with model stacking, (**E**) random forest model with model stacking, and (**F**) extreme gradient boosting benchmark model with model stacking. AVG = average; FAR = faricimab; LLD = low luminance deficit; M = male; Q4W = every 4 weeks; Q8W = every 8 weeks; RBZ = ranibizumab.

Figure S12 Best-corrected visual acuity regression: comparisons of true value versus predicted values for each model. Both predicted value and true value are in ETDRS letters. BM = benchmark; DNN = deep neural network; ETDRS = Early Treatment Diabetic Retinopathy Study; RF = random forest; MA = model averaging; MS = model stacking; XGB = extreme gradient boosting.

Figure S13 Binary variable of percent decrease in central subfield thickness from baseline threshold at 35% classification receiver operating characteristic curves calculated from the test set, for each model. BM = benchmark; DNN = deep neural network; RF = random forest; MA = model averaging; MS = model stacking; XGB = extreme gradient boosting.

Figure S14 Percent decrease in central subfield thickness from baseline classification confusion matrix calculated from the test set, for each model. The closest point to the top left corner in the receiver operator characteristics plot was chosen as the operating point. Act. = actual; BM = benchmark; DNN = deep neural network; RF = random forest; MA = model averaging; MS = model stacking; Pred. = predicted; XGB = extreme gradient boosting.
