# Supplementary material for: Machine Learning to Predict Faricimab Treatment Outcome in Neovascular Age-Related Macular Degeneration
Source: Ophthalmol Sci. 2023 Aug 18;4(2):100385. doi: 10.1016/j.xops.2023.100385 (PMC10585644; doi:10.1016/j.xops.2023.100385)
Supplement: Supplementary Materials [file mmc1.docx]

**Supplementary Materials**

Supplementary Methods

Prediction of Outcome Variables

SHapley Additive exPlanations (SHAP)^1^ is a game theoretic approach to explain the impact of each feature for individual predictions. Because the same classical ML models were used in benchmark models and model averaging, SHAP was applied to the benchmark models and model stacking. SHAP plots were generated to visualize the output of the models to assess the effect of baseline clinical and spectral domain OCT (SD-OCT) image variables on predicting best-corrected visual acuity (BCVA) and central subfield thickness (CST) reduction rate at month 9.

Supplementary Results

Impact of Clinical Features on Outcome Variables

SHAP plots revealed that baseline BCVA (Early Treatment Diabetic Retinopathy Study [ETDRS] letter score) had the biggest impact on BCVA regression at month 9 across all models (Fig S10). Predictions using DL models of SD-OCT B-scans had the second greatest impact in the model stacking approach.

For percent decrease in CST from baseline classification at month 9, baseline CST had the largest impact in all models (Fig S11); the SHAP plots show that patients with higher CST at baseline were more likely to experience reductions in CST of >35%. Baseline BCVA (ETDRS letter score) had the second and third greatest impact in the benchmark models and model stacking approach, respectively. Baseline BCVA had a negative impact, where a lower baseline BCVA contributed to a higher probability of achieving a reduction in CST of >35%. Predictions with DL models of SD-OCT B-scans had the second greatest impact on percent decrease in CST from baseline classification in the model stacking approach, but to a lesser extent than for BCVA regression.

In general, it was observed that random forest models used relatively more clinical features to make predictions, whereas linear and extreme gradient boosting models depended on relatively few clinical features (Figs S10 and S11).

Comparison of Predictions with True Data

For BCVA regression, the prediction varied in relation to the true value in terms of the final BCVA value at month 9 (Fig S12). For patients with very low BCVA at month 9 (20–40 letters), the predicted value was very different from the true value. In patients with a BCVA of 40–60 letters at month 9, predicted values were generally higher than true values. Conversely, predicted values were generally lower than true values in patients with a BCVA of >60 letters at month 9.

For percent decrease in CST from baseline classification, most models showed relatively high true positive rates (Fig S13, S14). However, the DL benchmark model indicated that predictions were close to random at higher rates, which reflects the lower AUROC values in this model (Table 4).

References

1. Lundberg SM, Lee S-I. A unified approach to interpreting model predictions. Presented at: 31st Conference on Neural Information Processing Systems (NIPS); 4-9 December 2017, 2017; Long Beach, CA.
