# Supplementary figures and images for: Machine Learning to Predict Faricimab Treatment Outcome in Neovascular Age-Related Macular Degeneration
Source: Ophthalmol Sci. 2023 Aug 18;4(2):100385. doi: 10.1016/j.xops.2023.100385 (PMC10585644; doi:10.1016/j.xops.2023.100385)

A

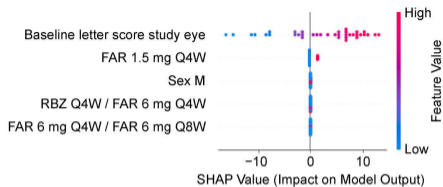

B

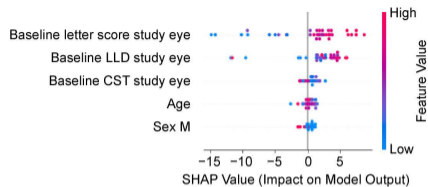

C

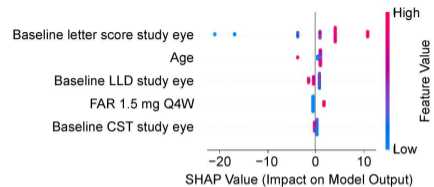

D

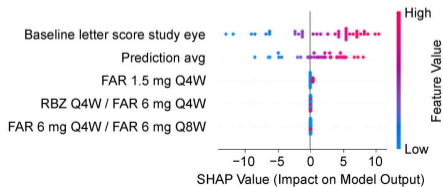

E

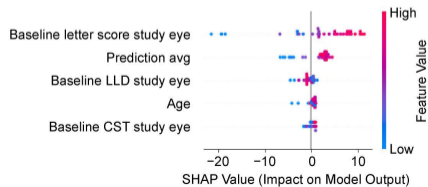

F

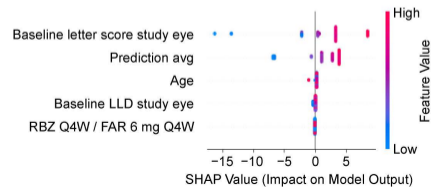

Supplement: Figure S10 — SHapley Additive exPlanations (SHAP) plots performed on the test set for best-corrected visual acuity regression at month 9 for (A) linear benchmark model, (B) random forest benchmark model, (C) extreme gradient boosting benchmark model, (D) linear model with model stacking, (E) random forest model with model stacking, and (F) extreme gradient boosting benchmark model with model stacking. Avg = average; CST = central subfield thickness; FAR = faricimab; LLD = low luminance deficit; M = male; Q4W = every 4 weeks; Q8W = every 8 weeks; RBZ = ranibizumab. [file mmc2.pdf]

A

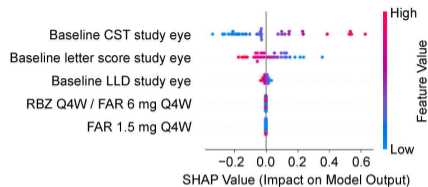

B

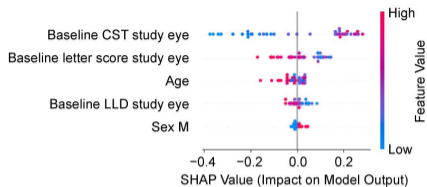

C

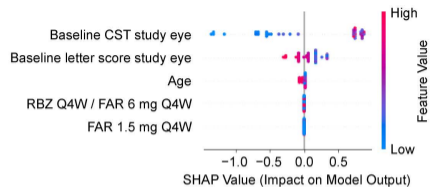

D

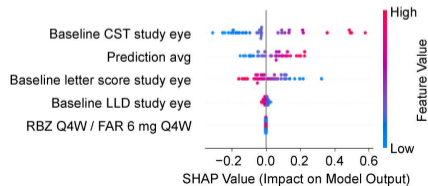

E

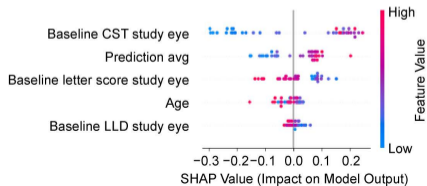

F

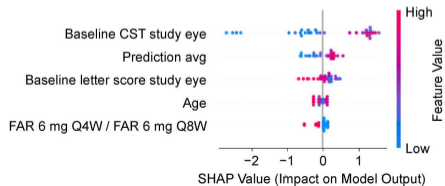

Supplement: Figure S11 — SHapley Additive exPlanations (SHAP) plots performed on the test set for percent decrease in central subfield thickness (CST) from baseline classification at month 9 for (A) linear benchmark model, (B) random forest benchmark model, (C) extreme gradient boosting benchmark model, (D) linear model with model stacking, (E) random forest model with model stacking, and (F) extreme gradient boosting benchmark model with model stacking. AVG = average; FAR = faricimab; LLD = low luminance deficit; M = male; Q4W = every 4 weeks; Q8W = every 8 weeks; RBZ = ranibizumab. [file mmc3.pdf]

Linear BM

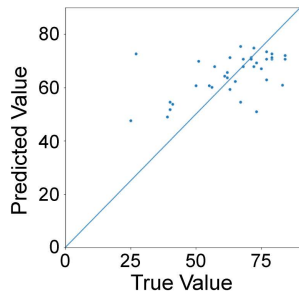

RF BM

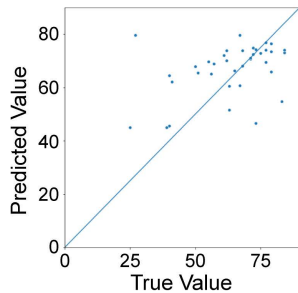

XGB BM

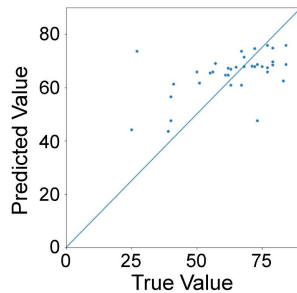

DNN BM

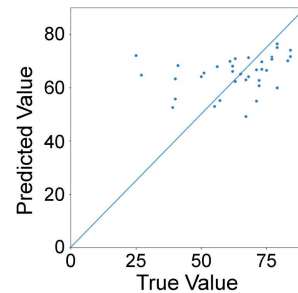

Linear MS

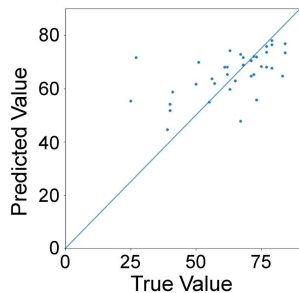

RF MS

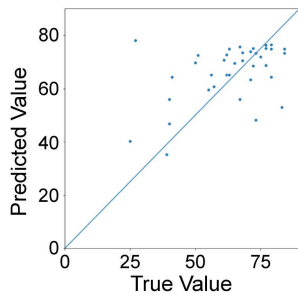

XGB MS

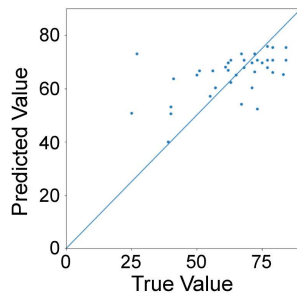

Linear MA

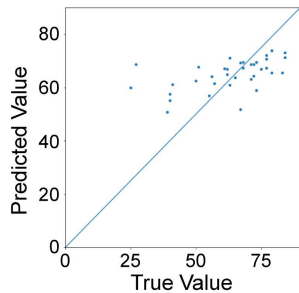

RF MA

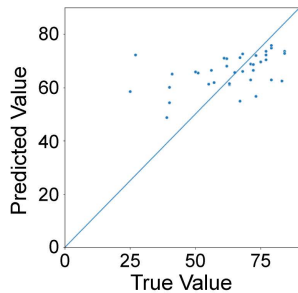

XGB MA

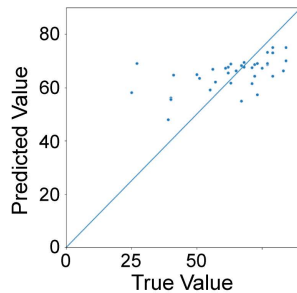

Supplement: Figure S12 — Best-corrected visual acuity regression: comparisons of true value versus predicted values for each model. Both predicted value and true value are in ETDRS letters. BM = benchmark; DNN = deep neural network; ETDRS = Early Treatment Diabetic Retinopathy Study; RF = random forest; MA = model averaging; MS = model stacking; XGB = extreme gradient boosting. [file mmc4.pdf]

Linear BM

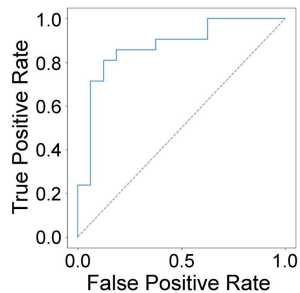

RF BM

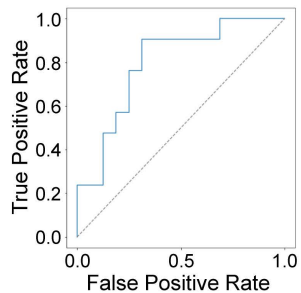

XGB BM

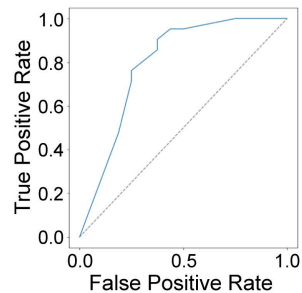

DNN BM

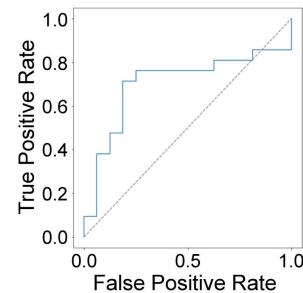

Linear MS

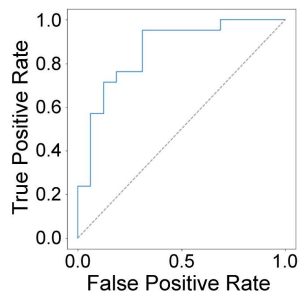

RF MS

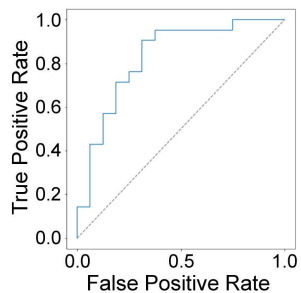

XGB MS

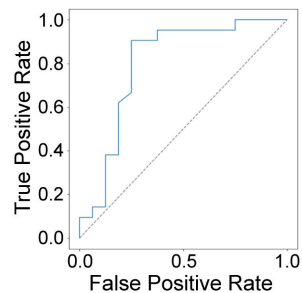

Linear MA

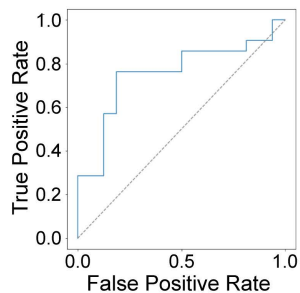

RF MA

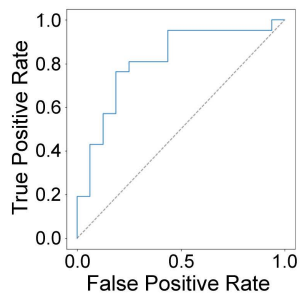

XGB MA

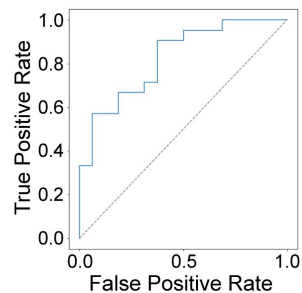

Supplement: Figure S13 — Binary variable of percent decrease in central subfield thickness from baseline threshold at 35% classification receiver operating characteristic curves calculated from the test set, for each model. BM = benchmark; DNN = deep neural network; RF = random forest; MA = model averaging; MS = model stacking; XGB = extreme gradient boosting. [file mmc5.pdf]
