# Supplementary material for: Machine Learning to Predict Faricimab Treatment Outcome in Neovascular Age-Related Macular Degeneration
Source: Ophthalmol Sci. 2023 Aug 18;4(2):100385. doi: 10.1016/j.xops.2023.100385 (PMC10585644; doi:10.1016/j.xops.2023.100385)
Supplement: Figure S14 — Percent decrease in central subfield thickness from baseline classification confusion matrix calculated from the test set, for each model. The closest point to the top left corner in the receiver operator characteristics plot was chosen as the operating point. Act. = actual; BM = benchmark; DNN = deep neural network; RF = random forest; MA = model averaging; MS = model stacking; Pred. = predicted; XGB = extreme gradient boosting. [file mmc6.pdf]

Linear BM

	Pred. Yes	Pred. No
Act. Yes	17	4
Act. No	2	14

RF BM

	Pred. Yes	Pred. No
Act. Yes	19	2
Act. No	5	11

XGB BM

	Pred. Yes	Pred. No
Act. Yes	19	2
Act. No	6	10

DNN BM

	Pred. Yes	Pred. No
Act. Yes	15	6
Act. No	3	13

Linear MS

	Pred. Yes	Pred. No
Act. Yes	20	1
Act. No	5	11

RF MS

	Pred. Yes	Pred. No
Act. Yes	19	2
Act. No	5	11

XGB MS

	Pred. Yes	Pred. No
Act. Yes	19	2
Act. No	4	12

Linear MA

	Pred. Yes	Pred. No
Act. Yes	16	5
Act. No	3	13

RF MA

	Pred. Yes	Pred. No
Act. Yes	16	5
Act. No	3	13

XGB MA

	Pred. Yes	Pred. No
Act. Yes	19	2
Act. No	6	10
